# Revisiting misfolding propensity of serum amyloid A1: Special focus on the signal peptide region

**DOI:** 10.1016/j.bbrep.2022.101284

**Published:** 2022-05-30

**Authors:** Morgan S. Haines, Eduardo Ramirez, Kendall B.E. Moore, Jessica S. Fortin

**Affiliations:** Department of Basic Medical Sciences, College of Veterinary Medicine, Purdue University, 625 Harrison Street, West Lafayette, IN, USA

**Keywords:** Amyloid A, Fibril assembly, Protein misfolding, Serum amyloid A, Signal peptide, Systemic amyloidosis, Agg, Aggregation, HFIP, Hexafluoroisopropanol, HDL, High-density lipoprotein, MMP, Metalloproteinases, SAA1, Serum amyloid A1, ThT, Thioflavin T, TEM, Transmission electron microscopy, Tris, Tris(hydroxymethyl)aminomethane

## Abstract

AA amyloidosis is the result of overproduction and aberrant processing of acute-phase serum amyloid A1 (SAA1) by hepatocytes. Proteolytic cleavage of SAA1 is believed to play a central role in AA amyloid formation. The SAA1 protein undergoes a cleavage of 18 residues consisting of the signal peptide at the N-terminal region. To better understand the mechanism behind systemic amyloidosis in the SAA1 protein, we studied the misfolding propensity of the signal peptide region. We first examined the signal peptide amino acid SAA derived from different animal species. A library of 16 peptides was designed to evaluate the propensity of aggregation. The amyloidogenic potential of each SAA1 signal peptide homolog was assessed using *in silico* Tango program, thioflavin T (ThT) fluorescence, transmission electron microscopy (TEM), and seeding with misfolded human SAA1 signal peptide. After 7 days of incubation, most of the SAA1 signal peptide fragments had the propensity to form fibrils at a concentration of 100 μM in 50 mM Tris buffer at 37 °C by TEM. All peptides were able to generate fibrils at a higher concentration, i.e 500 μM in 25 mM Tris buffer with 50% HFIP, by ThT. All SAA1 signal synthetic peptides designed from the different animal species had the propensity to misfold and form fibrils, particularly in species with low occurrence of systemic amyloidosis. The human SAA1 signal peptide region was capable to seed the SAA1 1–25 and 32–47 peptide regions. Characterizing fibrillar conformations are relevant for seeding intact and/or fragmented SAA, which may contribute, to the mechanism of protein misfolding. This research signifies the importance of the signal peptide region and its possible contribution to the misfolding of aggregation-prone proteins.

## Highlights

• Serum amyloid A1 (SAA1) signal peptide synthetic fragments have the propensity to form fibrils.
• SAA1 signal peptide, 1–25, and 51–75 regions are prone-to-aggregate.
• Human SAA1 signal peptide is capable to seeds human SAA1 1–25 and 32–47 fragments.
• SAA1 signal peptide fibrils may have a role in seeding intact and/or fragmented SAA1.

## Graphical abstract

Schematic representation of the hypothetical role of the signal peptide fragmentation on the mechanism of serum amyloid A1 (SAA1) misfolding. SAA1 is produced by hepatocytes and released in state of chronic inflammation. It has the propensity to misfold and form amyloid plaques in organs such as the liver, spleen, and kidney. The signal peptide region of SAA1 is cleaved. The mature SAA1 protein is further cleaved at the C-terminal region during or after the process of misfolding. Intracellularly, the pathways used to eliminate misfolding proteins might be overwhelmed. The cleaved signal peptide may reside long enough in the cell to misfold and seed further SAA1 fragments.

**Figure:**
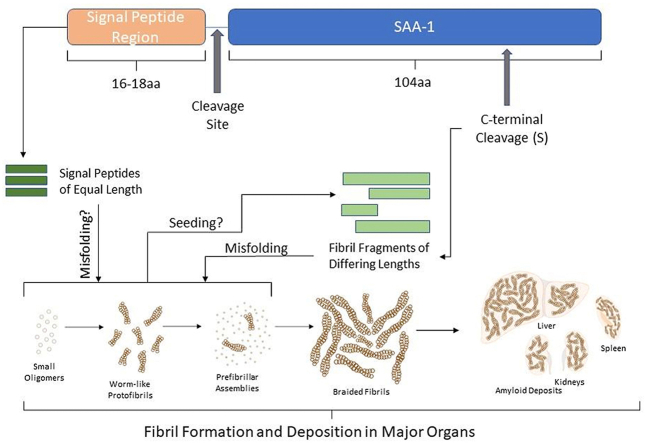

## 1 Introduction

AA amyloidosis is a common form of systemic amyloid disease, characterized by an accumulation of misfolded protein, reported in both human and veterinary medicine [1]. AA amyloid is derived from the positive acute-phase protein Serum Amyloid A (SAA), an apolipoprotein of high-density lipoprotein (HDL) [2]. AA amyloidosis is the result of the overproduction and aberrant processing of acute-phase SAA1 [3]. SAA1 dissociates from HDL through an interaction with heparan sulfate (glycosaminoglycan in the extracellular matrix) before its conversion to a fibrillary form [4]. SAA is produced by the liver during states of chronic inflammation [5,6]. SAA is encoded by four SAA genes in chromosome 11p15.1 in humans [7]. Despite sharing a strong nucleotide identity with SAA1, the C-terminally truncated SAA1 is predominantly found in amyloid deposits affecting organs such as the liver, spleen, and kidneys [8]. The accumulation of amyloid plaques over time may result in subsequent tissue damage and loss of function leading to organ failure.

Proteolytic cleavage of SAA1 is believed to play a central role in AA amyloid formation [7]. The SAA1 protein, which consists of 122 amino acids (aa) and a signal peptide at the N-terminal region, undergoes the first cleavage of 18 residues. This fracture results in a smaller protein of 104 aa (mature SAA1) [8]. Another cleavage of the mature SAA1 results in the first 76 residues, and other fragments have been detected in amyloid tissues [7]. The removal of the C-terminal tail results in a more unstable SAA1 peptide, prompting fibril formation [8]. SAA1 is cleaved by different metalloproteinases (MMP) that are induced by SAA during inflammation, including MMP-1, MMP-2, MMP-3 [9], MMP-9/Gelatinase-B [10], and MMP-10 [11]. Cleavage by MMPs generate one SAA1 fragment of 1–57 aa [9,12]. Fragments of various sizes have been observed, including a portion of 45–95 aa of SAA [[13], [14], [15], [16]]. It is uncertain if fragmentation occurs before or after fibril formation [8].

Through the use of a web-based prediction software known as AGGRESCAN [17] we detected one peptide region that would qualify as a “hot spot” in the SAA1 sequence, corresponding to the signal peptide region. Signal peptides are located at the N-terminal portion of proteins and determine their cellular localization. Simple modifications in signal peptide charges indicate different target locations of specific proteins [18]. The signal peptide aa structure consists of three parts: the N-, H-, and C-terminal regions, constituting a combination favorable for misfolding. The N-region harbors a positive charge. The H-region contains the hydrophobic portion of the signal peptide and forms an alpha helix. The C-region adopts a β-sheet secondary structure. The signal peptide is to be cleaved at the end of the C-region after the intracellular protein has been localized [19]. After its dissociation from the protein, the signal peptide fragment is believed to be eliminated after an indeterminate period of time [19]. Multiple studies seek to elucidate the physiological role of the signal peptide region. Previous studies have shown that the mutation of a protein's signal peptide has been linked to numerous diseases, such as hypoparathyroidism, diabetes, renal dysfunction, and coronary heart disease [20].

Our studies on protein misfolding propensity of the signal peptide region allow us to better understand the mechanism behind systemic amyloidosis in the SAA1 protein. To the best of our knowledge, the aggregation propensity of the SAA1 signal peptide region has never been studied *in vitro* using a small peptide library. The protein segment that is most prone to misfold may be in a region that is initially cleaved, and the resulting fragment peptides may adopt oligomeric and/or fibrillar conformations. It is not clear if protein misfolding occurs prior, during, and/or after cleavage. Characterizing these conformations is relevant as they may be involved in seeding intact and/or fragmented SAA1, contributing to the mechanisms of protein misfolding. Herein, we report the *in vitro* aggregation profiling of 16 fragment peptides representing the SAA1 signal peptide regions of human and various animal species.

## 2 Materials and methods

### 2.1 Chemicals and peptides

All signal peptides used in the study were prepared and obtained from GenScript USA Inc (Piscataway, NJ). Hexafluoroisopropanol (HFIP) and thioflavin T (ThT) were purchased from Alfa Aesar (Ward Hill, MA).

### 2.2 In silico analysis of fibril formation

SAA1 signal peptide sequences (17–18 amino acids) were sourced from NCBI based on the full SAA1 sequences of human and 22 different animal species (16 synthetic peptides in total). The aggregation propensity (aggregation score) of each signal peptide, as well as regions of the human SAA1, was predicted using *in silico* analysis with the Tango program [[21], [22], [23]].

### 2.3 Thioflavin T (ThT) fluorescence experiment with signal peptides

ThT fluorescence assays were performed with fragment peptides of the SAA1 signal peptide region to detect the formation of fibrils [[24], [25]]. Stock solutions of different SAA signal peptides were prepared with 100% HFIP or 50 mM Tris (pH 8) at a concentration of 1 mM. The non-treated black 96-well microplate with a transparent flat bottom (Corning, ref 3631) contained 50 μL of tris(hydroxymethyl)aminomethane (Tris) buffer (pH 8) (final concentration at 25 mM) and 50 μL of signal peptide (final concentration at 500 μM). ThT was added to produce a final concentration of 100 μM. The negative control (background signal) consisted of Tris buffer with ThT. The plate was sealed and fluorescence intensity was measured at 37 °C in a Synergy HT multi-mode microplate reader (BioTek, Winooski, VT). The excitation and emission wavelengths were set at 440 and 485 nm, respectively. Parameters of the plate reader consisted of slow shaking mode for 10 s prior to reading at 60 min intervals for 120 h. Two to three replicates of each signal peptide were assayed on the 96-well plate. ThT assays were repeated three times using two SAA fragment peptide stock solutions. The last five measurements of the sigmoidal curve were averaged and compared to the fluorescence background signal.

### 2.4 Transmission electron microscopy (TEM)

In a 1.5 ml microcentrifuge test tube, SAA1 signal peptides were incubated at 37 °C for 7 days. Peptide concentrations used were 100 μM and 500 μM using 50 mM Tris buffer (pH 8) or 50% HFIP in 25 mM Tris buffer. For peptides containing 50% HFIP, each tube was centrifuged at 14,000 rpm for 10 min prior to grid preparation. The supernatant was discarded and 100 μL of phosphate-buffered saline (pH 7.4) was added to remove the HFIP and preserve the grid integrity. For the peptide solubilized in 50 mM tris buffer (in the absence of HFIP), the centrifugation and wash steps were not applied. A small volume of the peptide solution (10 μL) was deposited on the grid. The grids utilized were 400-mesh Formvar-carbon-coated copper grids (Electron Microscopy Sciences, Hatfield, PA). Grids were washed three times with distilled water after an incubation of 1 min at room temperature. Water was removed with filter paper and grids were air-dried. A fresh solution of 1% uranyl acetate (1–2 μL) was deposited on each grid for 1 min. Visualization of the grids was performed with transmission electron microscopy (JEOL 1400 Flash, Japan). Acquisition of pictures was performed with accelerating voltage of 100 kV and magnification of 40 k.

### 2.5 Seeding experiment with SAA1 signal peptide 1 (human)

To prepare the seeds, the SAA1 signal peptide fragment 1 (human) was solubilized at 500 μM in 50% HFIP and 25 mM Tris buffer and incubated at 37 °C for 7 days. The sample was diluted at 100 μM in order to add 1 μL per well to obtain a final concentration of 1 μM of seeds. Seedings were achieved with prone-to-aggregate fragment 1–25 and less to null prone-to-aggregate fragment 32–47. The control consisted of the non-seeded peptide fragment. The experiment was performed using a 96-well plate containing one glass bead per well. For the non-seeded peptide fragment condition, we prepared 50 μL of 1 mM peptide fragment stock in 45 μL of 50 mM Tris Buffer (pH 8) and 5 μL of 2 mM ThT to give a final concentration of 500 μM signal peptide. For the seeded peptide fragment condition, we prepared 50 μL of 1 mM peptide fragment stock in 44 μL of 50 mM Tris Buffer (pH 8), 5 μL of 2 mM ThT, and 1 μL of 100 μM of SAA1 sample 1 (human) peptide to give a final concentration of 500 μM peptide fragment and 1 μM of SAA1 sample 1 (human) peptide. For the negative control (background signal), 5 μL of 2 mM ThT was added in 95 μL of 50 mM Tris Buffer (pH 8). The plate was sealed and fluorescence intensity was measured as described above in 2.3. For the analysis of data, 3 to 6 replicates of seeding/non-seeded condition were averaged together per peptide fragment and background fluorescence (control) was subtracted from the fluorescent readings of the peptide fragments. Plots containing the relative fluorescence intensity vs time (in hours) were created using Microsoft Excel for two of the peptide fragments in the seeded versus non-seeded condition.

## 3 Results

### 3.1 The signal peptide region of SAA1 of diverse species has high aggregation score

**Fig. 1:**
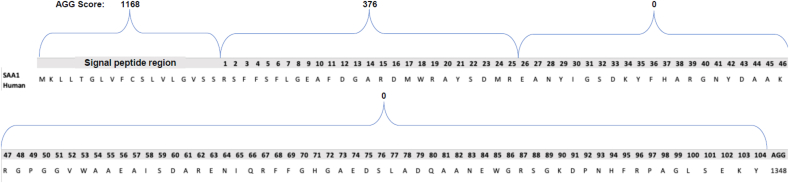
Aggregation (Agg) score results were obtained from different regions of the human serum amyloid A1 (SAA1). The highest Agg score resulted from the signal peptide region (1), and amino acids 1–25 (2), indicative of a high propensity for aggregation. Agg scores were obtained with the Tango program.

Human SAA1 amino acid sequence was selected as a positive control for all *in silico* analyses due to substantial reporting of AA amyloidosis in human clinical reports. Amino acids that diverged from the positive control are highlighted in red (Table 1). Aggregation (Agg) scores were obtained with Tango program, a computer algorithm capable of predicting the region of the protein most prone to aggregate [[21], [22], [23]]. 15 signal peptides exhibited an Agg score ranging from 845 to 1284. Aggregation signal peptide 25 had the lowest Agg score. Bioinformatic analysis of SAA1 signal peptide derived from multiple species indicated that the signal peptide of 17–18 aa (cleaved from the pre-protein) exhibited a higher propensity to aggregate. Within the protein of 104 aa, the Agg score of the proximal segment composed of the first 25 residues represented the second most prone-to-aggregate region (Fig. 1).

**Table 1:**
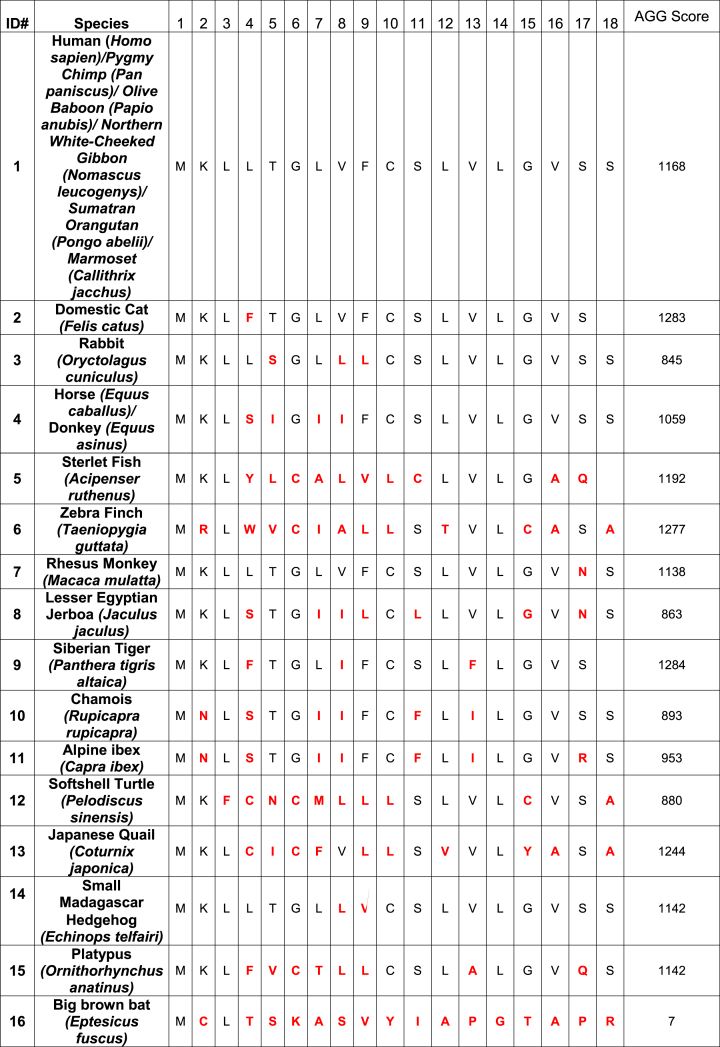
Peptide library of serum amyloid A1 (SAA1) signal peptide fragments composed of 17–18 amino acids. Sequences from species were obtained from Genbank. The red amino acids represent variations as compared to the human SAA1 signal peptide sequence. AGG: Aggregation.

### 3.2 Various synthetic fragment peptides representing different SAA1 signal regions form fibrils as monitored by ThT fluorescence assays

**Fig. 2:**
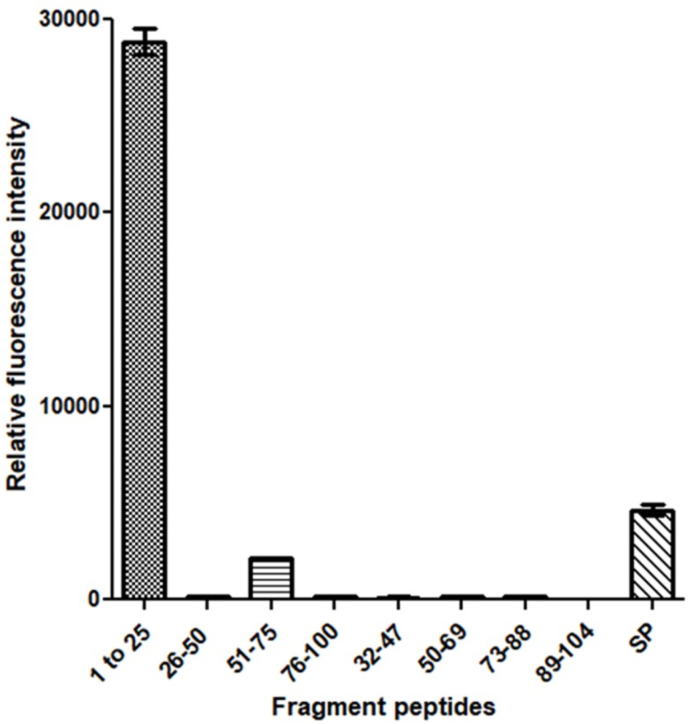
Comparison of thioflavin T (ThT) fluorescence intensity obtained at the end of fibrillization kinetics of different human SAA1 protein fragments at a concentration of 500 μM in 25 mM Tris buffer (pH 8) and 50% hexafluoroisopropanol (HFIP). Three samples of each fragment were monitored at 37 °C for 120 h.

The peptide library of 16 different fragments (of 17–18 residues) was synthesized by GenScript. Biophysical assays were performed to assess the aggregation propensity *in vitro* for each peptide to validate the aggregation score predictions via *in silico* analyses. Signal fragment peptides exhibiting high amyloidogenicity potential are likely to provide useful insights into the mechanisms underlying SAA1 fibril formation. ThT dye fluorescence was used to monitor *in vitro* fibril formation of each signal peptide. The fluorescent dye ThT specifically binds to the β-sheet structure of protein fibrils, providing a strong emission [[24], [25]]. The kinetic process of fibril formation of various SAA1 signal peptides was assessed at 500 μM with ThT fluorescence assays for 120 h at 37 °C. All peptides were present in the same plate. After testing the peptides at 500 μM, a histogram was generated from the average of the last five fluorescent values at the end of the kinetics of fibril formation (plateau phase).We first evaluated the aggregation propensity of 9 different regions of the human SAA1 peptide: 1–25, 26–50, 51–75, 76–100, 32–47, 50–69, 73–88, 89–104, and signal peptide (SP). The maximum fluorescence intensity obtained with the ThT assays was compared for each peptide fragment representing different regions of human SAA1 (Fig. 2). Fragment 1–25 exhibited the highest fluorescence intensity, followed by the signal peptide region and fragment 51–75 (Fig. 2).

**Fig. 3:**
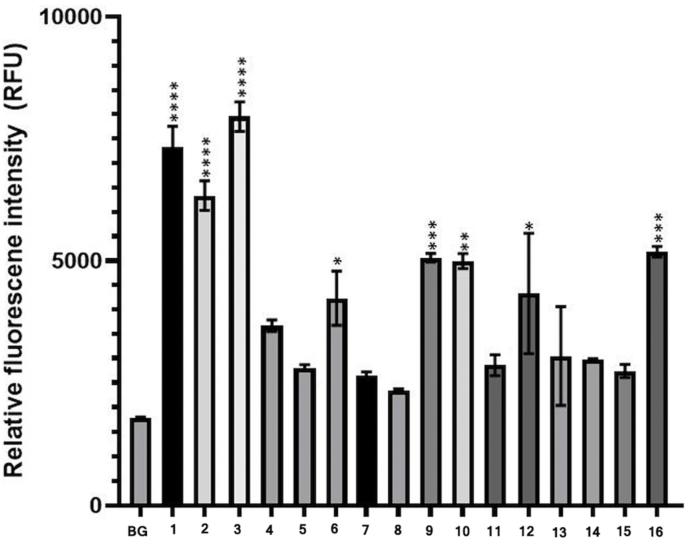
Thioflavin T (ThT) fluorescence emission in the presence of different serum amyloid A1 (SAA1) signal peptides. Signal peptides were solubilized at 500 μM in 25 mM Tris buffer (pH 8) and 50% hexafluoroisopropanol (HFIP). ThT-fluorescence was monitored at 37 °C for 120 h. ThT was used to create a final concentration of 100 μM. Each bar represents the average of the last five fluorescence values obtained. Error bars represent the standard error of the mean (SEM). Data were analyzed by the ordinary one-way ANOVA with Dunnett's multiple comparisons post-hoc testing between each SAA1 signal peptide and fluorescence background signal (BG). (Significance difference: *p<0.02, **p<0.001, ***p<0.0008, ****p<0.0001).

**Fig. 4:**
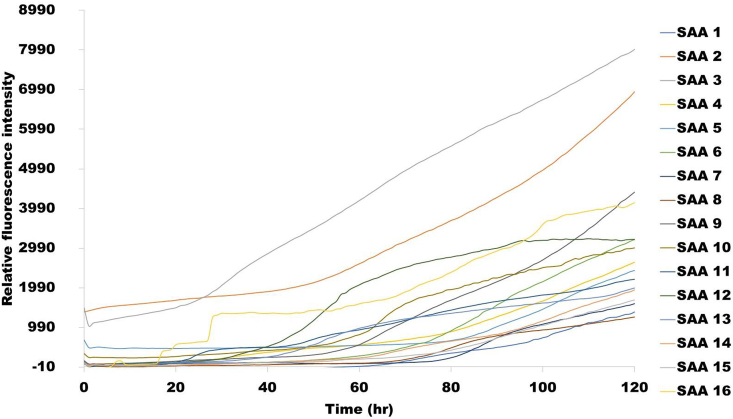
Kinetics of 16 different SAA1 signal peptide aggregations at 37 °C probed by thioflavin T (ThT) fluorescence. The 16 peptides represent the signal peptide region of different animal species. Signal peptides were solubilized at 500 μM in 25 mM Tris buffer (pH 8) and 50% hexafluoroisopropanol (HFIP). Each curve represents an average of the fluorescence intensity obtained from three replicates over 120 h (5 days) in incubation at 37 °C.

We then studied different signal peptide regions of SAA1 amino acid sequences from various animal species to validate the propensity to misfold in animals with a high and low occurrence of systemic amyloidosis. Fig. 3 shows the different relative fluorescence intensity by comparison with the fluorescence background signal (BG). SAA1 signal peptide 1 (human), 2 (cat), 3 (rabbit), 6 (zebra finch), 9 (amur tiger), 10 (chamois), 12 (soft-shelled turtle) and 16 (brown bat) all had statistically significant higher ThT fluorescence emissions. The kinetic aggregation curves (with subtracted background fluorescence) are shown in Fig. 4. All peptides exhibited some degree of sigmoidal- and exponential-like curves, which may support the misfolding of these signal peptide fragments designed from the amino acid sequences of various animal species. However, direct visualization of fibrils with a non-dye methodology was necessary to confirm the misfolding of SAA1 signal peptides resulting in non-statistically significant increase level of ThT fluorescence (indicated in Fig. 3).

### 3.3 The fibrillar morphology of SAA1 signal peptides from different animal species is assessed by transmission electron microscopy (TEM)

**Fig. 5:**
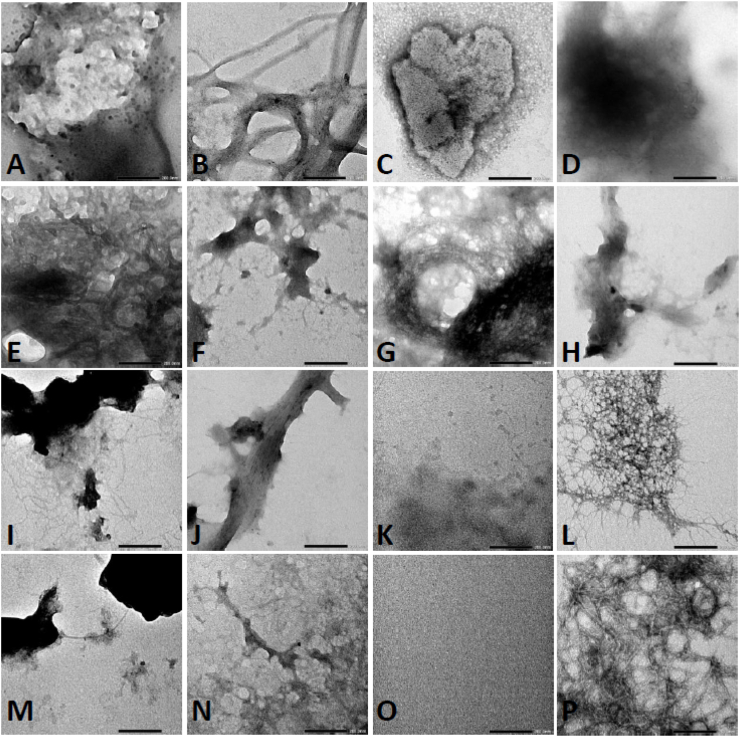
Representative images of serum amyloid A1 (SAA1) signal peptide library obtained by transmission electron microscopy (TEM). Signal peptides were solubilized at 100 μM in 50 mM Tris buffer and incubated at 37 °C for 7 days. (A) SAA1 signal peptide ID# 1 (human). (B) SAA1 signal peptide ID# 2 (cat). (C) SAA1 signal peptide ID# 3 (rabbit). (D) SAA1 signal peptide ID# 4 (horse). (E) SAA1 signal peptide ID# 5 (sterlet fish). (F) SAA1 signal peptide ID# 6 (zebra finch). (G) SAA1 signal peptide ID# 7 (rhesus monkey). (H) SAA1 signal peptide ID# 8 (lesser jerboa). (I) SAA1 signal peptide ID# 9 (amur tiger). (J) SAA1 signal peptide ID# 10 (chamois). (K) SAA1 signal peptide ID# 11 (alpine ibex). (L) SAA1 signal peptide ID# 12 (soft-shelled turtle). (M) SAA1 signal peptide ID# 13 (Japanese quail). (N) SAA1 signal peptide ID# 14 (hedgehog). (O) SAA1 signal peptide ID# 15 (platypus). (P) SAA1 signal peptide ID# 16 (brown bat). A scale bar of 200 nm is indicated in the right bottom corner of each photomicrograph.

**Fig. 6:**
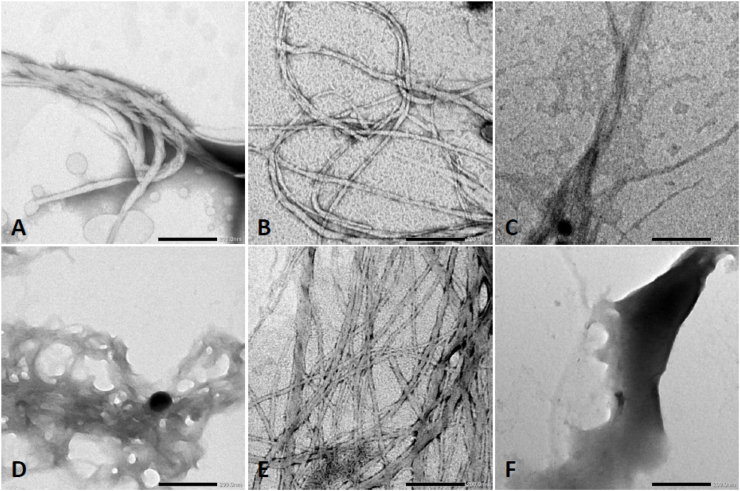
Representative images of serum amyloid A1 (SAA1) signal peptide library obtained by transmission electron microscopy (TEM). Signal peptides were solubilized at 500 μM in 25 mM Tris buffer with 50% hexafluoroisopropanol (HFIP) and incubated at 37 °C for 7 days. (A) SAA1 signal peptide ID# 1 (human). (B) SAA1 signal peptide ID# 3 (rabbit). (C) SAA1 signal peptide ID# 4 (horse). (D) SAA1 signal peptide ID# 11 (alpine ibex). (E) SAA1 signal peptide ID# 13 (Japanese quail). (F) SAA1 signal peptide ID# 15 (platypus). A scale bar of 200 nm is indicated in the right bottom corner of each photomicrograph.

To confirm the presence of fibrils, the morphology of SAA1 signal peptides was first examined after 7 days of incubation at 37 °C. Peptides were solubilized at 100 μM in 50 mM Tris buffer (pH 8). Fibrils were detected on grids containing SAA1 signal peptides 2 (cat), 5 (sterlet fish), 6 (zebra finch), 7 (rhesus monkey), 8 (lesser jerboa), 9 (amur tiger), 10 (chamois), 12 (soft-shelled turtle), 14 (hedgehog) and 16 (brown bat) (Fig. 5). SAA1 signal peptides 2 (cat), 5 (sterlet fish), 7 (rhesus monkey), and 14 (hedgehog) resulted in a dense mat of intertwined fibrils. Entangled thinner fibrils were observed with SAA1 signal peptides 12 (soft-shelled turtle) and 16 (brown bat). Combined thick fibrils were noted with signal peptides 2 (cat), 5 (sterlet fish), 7 (rhesus monkey), and 14 (hedgehog). Thin fibrils were present on the edge of the plaque-like materials for SAA1 signal peptides 6 (zebra finch), 8 (lesser jerboa), 9 (amur tiger), 10 (chamois), and 13 (Japanese quail). Signal peptide 13 (Japanese quail) was prepared at a higher concentration to confirm the presence of fibrils (Fig. 6). Various degrees of porous materials were detected on the grids of peptide 1 (human) and peptide 3 (rabbit). Grids prepared with SAA1 signal peptides 1 (human), 3 (rabbit), 4 (horse), 11 (alpine ibex), 13 (Japanese quail), and 15 (platypus) did not contain fibrils. These fragment peptides were processed at a higher concentration, i.e. 500 μM, using 50% HFIP and 25 mM Tris buffer, to detect the presence of fibrils. Photomicrographs are shown in Fig. 6.

The SAA1 signal peptides deemed negative at 100 μM were studied at higher concentration. At a concentration of 500 μM using 25 mM Tris buffer and 50% HFIP, all peptides formed fibrils. SAA1 signal peptides 1 (human), 3 (rabbit), and 13 (Japanese quail) adopted extensive long linear fibrillar structures. Thick combined twisted fibrils were noted in presence of signal peptide 1 (human). Shorter entangled fibrils were observed with signal peptide 11 (alpine ibex) (Fig. 6). Linear and short fibrils were detected with signal peptide 4 (horse). Signal peptide 15 (platypus) resulted in plaque-like deposited materials, presumably made of tightly packed fibrils with the thinnest fibrils congregating at the edge.

### 3.4 The human SAA1 signal peptide is capable to seed other fragmented regions of SAA1

**Fig. 7:**
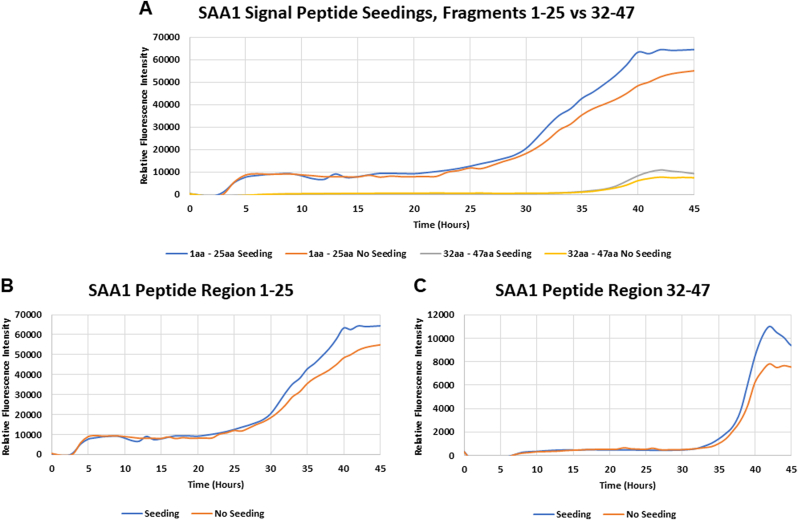
Seeding effect of preformed human SAA1 signal peptide aggregates on the kinetics of the 1–25 and 32–47 fragmented regions of human SAA1. (**A**) depicts a comparison of the fluorescence intensity between the aggregation kinetics of fragments 1-25 and 32-47. The graphs below portray time course of human SAA1 fragment peptide 1–25 (B) and 32–47 (C) fibril formation induced by incubation at 37 °C in the presence or absence of 1 μM misfolded human SAA1 signal peptide (seeds). Aggregation kinetics were monitored with thioflavin T (ThT). Fragment peptides 1–25 and 32–47 were prepared at 500 μM in 25 mM Tris buffer (pH 8) and 50% hexafluoroisopropanol (HFIP).

The SAA1 peptides containing the 1 – 25 and the 32 – 47 fragmented regions were subjected to seeding with SAA1 signal peptide ID# 1 (human) in order to analyze the aggregation propensity of the SAA1 peptide. When neither peptide fragment was in contact with misfolded SAA1 signal peptide (seeds), the ThT fluorescence intensity steadily increased over time (Fig. 7A: No Seeds) indicating gradual fibrillization of SAA1 fragments. In the absence of seeds, the fluorescence intensity is higher with fragment 1–25 in comparison to fragment 32–47 (Fig. 7A). This result is consistent with data presented in Fig. 2. When the two peptide fragments are in contact with SAA1 human signal peptide seeds, the ThT fluorescence intensity increases over time after about 25 to 35 h at a greater magnitude than the non seeded peptides (Fig. 7B-C: Seeding).

## 4 Discussion

We report herein that the signal peptide region of SAA1 can misfold. In addition to the resulting peptide fragments, we demonstrated that the signal peptide region is capable to adopt fibrillar conformation, which can act as a template to seed 1–25 and 32–47 fragment peptides. The SAA1 signal peptide fragments included in this study with the aggregation score obtained from the bioinformatics program are summarized in Table 1. Data were obtained with 16 SAA1 signal peptides resourced from NCBI search on SAA1 aa sequences of human and animal species. Signal peptides considered amyloidogenic are expected to be positively detected by ThT and TEM [26,27]. The *in silico* analysis of the SAA1 signal peptides showed all but one peptide having aggregated. The aggregation score of peptide 16 (brown bat) scored a 7, but the synthetic fragment peptide was capable to generate fibrils as assessed by ThT assays (Fig. 3, Fig. 4) and TEM (Fig. 5). The rest of the SAA1 signal peptide regions scored at a range from 845 to 1284, with all signal peptide fragments resulting in positive TEM (Fig. 5, Fig. 6). *In silico* analysis of these peptides was useful to identify the SAA1 signal peptide region as a "çhot spot", but not necessarily suitable to determine peptide-specific amyloidogenicity of this particular region. Such put an emphasis on performing ThT assays, TEM analyses, and seeding experiments.

According to the TEM, most SAA1 peptides formed fibrils at 100 μM using 50 mM Tris buffer (pH 8). For the five negative samples, stock solutions were assayed at higher concentration (500 μM) using 50% HFIP in 25 mM Tris buffer. A high concentration of HFIP allows for the prevention of hydrogen bonding [24]. This mechanism may prevent amyloidogenic proteins from folding into β-sheets for some peptides [28]. HFIP accelerates kinetic fibril formation when used at a lower concentration. We used two different systems of buffer to ensure peptides were soluble at high concentration and to provide different pH. Intracellularly, pH may vary depending on the subcellular localization of the SAA1 signal peptide. For example, ongoing lysosomal trafficking will lower pH. In our *in vitro* studies, high concentrated peptide (i.e. 500 μM) requires 50% HFIP and all signal peptides were misfolded at low pH. At low concentration (i.e. 100 μM) in 50 mM of Tris buffer, 11 signal peptide demonstrated fibrillar structures by TEM.

ThT based-dye assays were first utilized to monitor fibril formation and the presence of fibrils was confirmed with a direct method, i.e. TEM. Concerning the ThT assays, a concentration of 100 μM of buffer was not adequate to monitor fibril formation. Misfolded signal peptides were detected at 100 μM by TEM (Fig. 5). For the samples deemed negative at 100 μM, the concentration of the 500 μM confirmed the ability of fibril formation (Fig. 6). A high concentration of protein may favor the intermolecular interactions and increase the ability for fibril formation. For the TEM analysis, a 7-day incubation period at 37 °C was required to allow for the formation of fibrils.

Synthetic SAA1 signal peptides were designed from amino acid sequences of diverse animal species to correlate the experimental amyloidogenicity with the occurrence of systemic amyloidosis described in case reports. Several of the SAA1 signal peptides studied come from the amino acid sequence of reptiles and fish where no cases of systemic amyloidosis have been recorded: for example, Sterlet Fish (*Acipenser ruthenus*) (signal peptide 5) and Chinese Softshell Turtle (*Pelodiscus sinensis*) (signal peptide 12) [29]. SAA1 signal peptide region from all aforementioned species have been confirmed through this experiment to form fibrils. Amyloidosis research in reptiles and fish has been brief and limited; however, reptiles and fish have low propensity to aggregate systemic amyloidoisis (due to misfolding SAA1). The SAA1 signal peptide region propensity to misfold may be not dependent on the amino acid variation observed across different animal species (no phylogenetic dependence). Other conditions such as the concentration of the SAA1 protein (particularly if overexpressed), inflammatory state, immune system, and/or the presence of reactive oxygen species (ROS) may contribute to provide the ideal milieu for the misfolding of SAA1 protein. The analysis of 16 SAA1 signal peptide fragments provided a strong pool of samples to validate the propensity of the SAA1 signal peptide region to misfold.

This project identified the SAA1 signal peptide fragment that possesses the ability to form fibrils *in vitro*. The SAA1 protein is post-translationally changed by cleavage and resulting fragments are found in amyloid deposits. The fragmentation process may have importance in the overall protein stability, and the fragments generated (i.e. signal peptide region) have depicted fibril formation herein. The seeding behavior of the SAA1 signal peptide region was assessed. Results demonstrate that misfolded human SAA1 signal peptide fragment is capable to act as a for what template and seeds what do they do with high (e.g. fragment peptide 1–25) and low (e.g. fragment peptide 32–47) propensity to aggregate fragments (Fig. 7). Peptide fragments 1–25 and 32–47 resulted in a higher level of fluorescence intensity when seeded, i.e. in contact with misfolded human SAA1 signal peptide. This study demonstrated the seeding behavior of human SAA1 signal peptide fragments; further studies are needed to provide insightful and clinically relevant means. Further functional studies will provide mechanistic insights into the pathogenic contribution of protein regions not previously explored in SAA1-mediated AA amyloid formation. In this study, the SAA1 signal peptide region from human and a variety of animal species generated fibrils *in vitro,* with all of them confirmed by TEM. Our study underlines the possible involvement of the signal peptide region in the misfolding of SAA1, opening doors to the assessment of this region with other prone-to-aggregate proteins.

## Credit author statement

Morgan S. Haines: Data curation, Formal analysis, Validation, and Original draft writer. Eduardo Ramirez: Data curation, Formal analysis, Methodology, Investigation, and Validation. Kendall B. E. Moore: Editing, Validation, Conceptualization of **Graphical abstract**. Jessica S. Fortin: Conceptualization, Data curation, Investigation, Supervision, Writing-review, and Editing.

## Funding sources

This project was supported by the American Society for Investigative Pathology (ASIP) Summer Research Opportunity in Pathology Program (SORPP).

## Data Availability

No data was used for the research described in the article.
