# Corrigendum to “Revisiting misfolding propensity of serum amyloid A1: Special focus on the signal peptide region” [Biochem. Biophys. Rep. 31 (2022) 1–9/101284]

**DOI:** 10.1016/j.bbrep.2024.101775

**Published:** 2024-07-16

**Authors:** Morgan S. Haines, Eduardo Ramirez, Kendall B.E. Moore, Jessica S. Fortin

**Affiliations:** Department of Basic Medical Sciences, College of Veterinary Medicine, Purdue University, West Lafayette, IN, 47906, United States

The authors regret some minor mistakes that have occurred in the amino acid sequences of the signal peptide region. This does not change the general conclusion pertaining to human SAA signal peptide sequence and all others that were studied during this special investigation. In the signal peptide sequences of certain species listed in, we identified inaccuracies has detailed as follows.

1. **Marmoset:** In the original paper, amino acid 17 in the signal peptide was incorrectly identified as serine. It should be corrected to histidine.
2. **Domestic Cat, Sterlet Fish, and Siberian Tiger:** In the original paper, the signal peptide region for these species was stated as ending at amino acid 17, but it should extend from amino acid 1 to 18.
3. **Rabbit:** In the original paper, the signal peptide region for the rabbit was indicated as ending at amino acid 18; however, it should span from amino acid 1 to 19.

The marmoset shares a signal peptide sequence with the human (represented as ID #1 in Table 1). Due to the error in amino acid 17, we recommend removing the marmoset from the paper.

To rectify the errors for the other four species, we have recalculated the Tango scores with the correct amino acid sequences.

• **Domestic Cat**: The corrected aggregation score is 1257.04, differing by −25.96 units from the aggregation score of 1283 in the paper.
• **Rabbit**: The corrected aggregation score is 784.573, differing by −60.427 units from the aggregation score of 845 in the paper.
• **Sterlet Fish**: The corrected aggregation score is 1115.58, differing by −76.42 units from the aggregation score of 1192 in the paper.
• **Siberian Tiger**: The corrected aggregation score is 1257.36, differing by −26.64 units from the aggregation score of 1284 as listed in the paper.

The authors would like to apologise for any inconvenience caused.

